# Rapid regrowth and detection of microbial contaminants in equine fecal microbiome samples

**DOI:** 10.1371/journal.pone.0187044

**Published:** 2017-11-01

**Authors:** Kalie F. Beckers, Christopher J. Schulz, Gary W. Childers

**Affiliations:** Department of Biological Sciences, Southeastern Louisiana University, Hammond, LA, United States of America; Wageningen University, NETHERLANDS

## Abstract

Advances have been made to standardize 16S rRNA gene amplicon based studies for inter-study comparisons, yet there are many opportunities for systematic error that may render these comparisons improper and misleading. The fecal microbiome of horses has been examined previously, however, no universal horse fecal collection method and sample processing procedure has been established. This study was initialized in large part to ensure that samples collected by different individuals from different geographical areas (i.e., crowdsourced) were not contaminated due to less than optimal sampling or holding conditions. In this study, we examined the effect of sampling the surface of fecal pellets compared to homogenized fecal pellets, and also the effect of time of sampling after defecation on ‘bloom’ taxa (bloom taxa refers to microbial taxa that can grow rapidly in horse feces post-defecation) using v4 16S rRNA amplicon libraries. A total of 1,440,171 sequences were recovered from 65 horse fecal samples yielding a total of 3,422 OTUs at 97% similarity. Sampling from either surface or homogenized feces had no effect on diversity and little effect on microbial composition. Sampling at various time points (0, 2, 4, 6, 12 h) had a significant effect on both diversity and community composition of fecal samples. Alpha diversity (Shannon index) initially increased with time as regrowth taxa were detected in the amplicon libraries, but by 12 h the diversity sharply decreased as the community composition became dominated by a few bloom families, including *Bacillaceae*, *Planococcaeae*, and *Enterococcaceae*, and other families to a lesser extent. The results show that immediate sampling of horse feces must be done in order to ensure accurate representation of horse fecal samples. Also, several of the bloom taxa found in this study are known to occur in human and cattle feces post defecation. The dominance of these taxa in feces shortly after defecation suggests that the feces is an important habitat for these organisms, and horse fecal samples that were improperly stored can be identified by presence of bloom taxa.

## Introduction

The gut microbiota are viewed as an organ that plays a central role in host physiology, nutrition, immunomodulation, protection from pathogens, and disorders of the gastrointestinal tract of humans [1,2]. The nutrition and health of horses is also closely tied to their gastrointestinal microbiota [3]. Horses are herbivores and hindgut fermentors, which rely on their gut microbiota to break down plant structural carbohydrates that are not digestible by the host. The microbiota also produce short-chain volatile fatty acids that can provide maintenance energy [4–7]. Domestication has altered the natural feeding habits of horses, resulting in gastrointestinal disorders as the major cause of mortality and morbidity [8]. For example, diets high in starch, fructans, or simple sugars can lead to excess lactic acid production which may result in laminitis or colic [3,9,10]. The addition of oats (high in starch) to a hay diet has been shown to alter both the microbiota composition and function [11]. In addition to diet, use of antimicrobials [12], intense exercising and aerobic conditioning [13], and possibly geography, breed, husbandry, seasonal effects may play roles in shaping the horse gut microbiota [14].The intricate relationship between horse health and gut microbiota has been motivation for Next Generation Sequencing (NGS) enabled investigations of the equine gut microbiota [11,15–18].

The NGS revolution has given scientists new tools to examine the ecological, metabolic, and evolutionary associations between host organisms and their microbiota. One of the more attractive features of NGS microbiome studies is the potential for direct data comparison across multiple studies, which may lead to new hypotheses. Key obstacles to microbiome cross-study comparisons such as standardization of sample and metadata collection and lab protocols have previously been addressed by the Human Microbiome Project as a starting point. Subsequently, adopted methods were put forth by the Earth Microbiome Project as standard protocols that could enhance future comparisons between samples collected and processed by different labs [19]. Additionally, requirements by journals that NGS data is deposited into archives helps ensure that differences in bioinformatic and statistical methods between studies can be overcome by allowing direct access to raw data. Even with standardized protocols, systematic sources of error can still find their way into NGS studies. One step that can lead to large systematic bias in horse fecal microbiome studies is choice of DNA extraction methods, which has been shown to alter the ratio of *Firmicutes* and *Bacteroidetes* in equine fecal 16S amplicon libraries [20] and suitability of DNA for PCR and downstream NGS [21]. Bead beating time and force has a particularly strong effect on the composition of DNA obtained [22]. Reagent and laboratory contamination can compromise results of NGS based microbiome studies, especially in low microbial biomass samples or when little sample DNA is used [23], and must be dealt with to avoid drawing false conclusions. Another potential source of systematic error in NGS studies is sample collection and storage methods [24–26]. While it is widely accepted that immediate freezing at -80°C is the best practice for fecal sample preservation, numerous recent studies have demonstrated that alternative methods are available when -80°C is not possible or practical [26–31]. Even with advances in sample storage techniques, the surge in crowdsourcing of microbiome projects presents the problem of samples being in transit for varying amount of time and differences between sampling techniques of participants. The American Gut Project (AGP), which is the largest crowdsourced microbiome project to date, encountered a problem that some samples were found to have an overabundance of *Enterobacteriaceae*, *Psuedomonas* spp., *Bacillales*, and *Lactobacillus* spp., presumably due to regrowth, or ‘blooming’ of these taxa during transit. The AGP addressed this issue by use of a bloom filter, which removes sequences identified as matches to the taxa that are thought to bloom at ambient conditions post defecation (American Gut Project GitHub page, accessed 7/1/2016 at https://github.com/biocore/American-Gut/tree/master/data/AG/BLOOM.fasta). These methods have recently been described to correct for blooms that occur during sample handling and transport of human fecal samples [32]. While the identification of human fecal bloom taxa is crucial for studies such as the AGP, it is unclear, and perhaps doubtful that a human derived bloom filter and contaminant detection strategy is appropriate for non-human fecal microbiome studies.

In addition to obtaining basic information on horse fecal bloom contaminants, knowledge of the timing that bloom occurs would be useful to assess the possibility of collecting fecal samples from horses housed in stalls without having to directly observe time of defecation so long as a time-window is known. Being able to identify possible ‘bloom’ taxa in horse fecal samples would give greater confidence to crowdsourced projects where horse fecal samples can be collected at many different locations and processed in a single laboratory using a standard lab protocol, avoiding many potential biases.

We hypothesized that horse feces would likely undergo shifts in microbial community composition due to blooms of taxa, and further hypothesized that these bloom taxa may not be the same as those found in the AGP, since the human and horse fecal microbiome have different compositions. We also sought to determine if there is an effect of sampling from the surface of horse fecal pellets vs. homogenizing of fecal pellets with regards to microbial community composition estimation. Thus, the objectives of this study were to examine the technical reproducibility of two collection methods, surface fecal pellet sampling vs. homogenized fecal pellet sampling, and to investigate the identities and timing of potential bloom taxa in horse fecal samples using NGS. The main objectives of this study are (i) examine the technical reproducibility of 16S rRNA-based NGS surveys of the horse fecal microbiome using two different sampling techniques (surface sampling of manure, or homogenization of manure), (ii) track the changes in horse fecal microbiome post deposition from fresh to 12 h and identify horse fecal regrowth (i.e., bloom) taxa, and (iii) examine the feasibility of sampling horses using the wait-and-watch-for-the-drop technique (i.e., fresh collection) vs. collection of less than six h fecal samples from stalls by a pilot field study. With these data we hope to aid research of the horse fecal microbiome by addressing the effects of sampling and providing a potential method for identifying horse fecal samples that have bloom contamination.

## Materials and methods

All samples collected in this study were collected post-defecation and animals were not housed at Southeastern Louisiana University or manipulated in any way as part of this research project, including diet or otherwise. All animals were cared for on private farms and were treated humanely.

### Study sites, fecal sample collection, and sample storage

Horse *(Equus caballus*) fecal samples were collected from three privately owned farms in Southeast Louisiana with permission from owners. Farm #1 (Hammond) is located in Hammond, LA, USA. Farm #2 (Loranger) is located in Loranger, LA, USA. and Farm #3 (Folsom) is located in Folsom, LA, USA. A List of all samples and their associated metadata (farm, age, gender, diet, etc.) can be found in S1 Table.

Horse fecal sampling for each objective is detailed with graphical flow charts in S1 Protocol. For objective **(i)** three freshly deposited manure piles from three individual horses were visually observed at the time of deposition at the Loranger Farm, and sampling was performed immediately afterwards by removing small (approx 2g) scrapings from the exterior of a single pellet for ‘surface’ and placing into separate sterile 36 oz whirl-paks (Nasco, Inc. Fort Atkinson, WI, USA). Following ‘surface’ sampling, the remaining fecal pellet was placed into a whirl-pak for ‘homogenized’ sampling, and all samples were placed on ice and transported directly to a -20°C freezer until DNA extraction, for a total of 18 samples. After samples were thawed and immediately before DNA extraction, ‘homogenized’ samples were homogenized by kneading the pellet inside the whirl-pak by hand for 1min, then DNA extraction proceeded as described below. For objective **(ii),** three fresh manure piles were marked off at time of deposition from three individual horses at the Loranger Farm and five samples were taken at timed intervals (14 samples total, one lost in processing) from each manure pile. All samples were in shaded areas (barn). The average ambient temperature for the 12 h period was 32°C (SD ± 3.6). At time of deposition (T0) an individual pellet from the manure pile was collected, immediately frozen, then processed using the homogenized sampling method. Additional samples were collected at 2, 4, 6, and 12 h (T2, T4, T6, and T12). To address objective **(iii)** 24 samples were collected, six samples from the Loranger Farm, five samples from the Hammond Farm, and 13 samples from the Folsom Farm in January of 2015. All samples from the Hammond and Loranger Farms were collected by visually observing a natural defecation event, collected in sterile whirl-paks, transported on ice to a -20°C freezer, and processed using the ‘homogenized’ technique. Samples collected from the Folsom Farm were collected from individual stalls that housed a single horse with a clay surface with wood shaving. The stalls had been cleaned within the previous six hours by removal of horse and wood shavings, and addition of new wood shavings. Samples were processed using the homogenized sampling procedure.

### DNA extraction

Microbial DNA was extracted from fecal samples using the MoBio PowerSoil® DNA Isolation kit (Mo Bio Laboratories Inc., Carlsbad, CA). Samples were thawed at room temperature and approx 0.25g of each sample was added directly to the PowerBead Tubes while samples were still cold to touch. DNA extraction was performed according to manufacturer’s protocol.

### PCR amplification of the V4 region of the 16S rRNA gene and NGS

The V4 variable region of 16S rRNA encoding gene was amplified using 515F and 806R fusion primers [33] suitable for the Ion Torrent PGM workflow. The fusion primers contained Ion Primer Adapter A on the 5’ end, followed by a unique 10 bp Hamming-error-correcting barcode [34], a 3 bp ‘GAT’ spacer, and the 515F primer sequence to allow for multiplexing of samples. The reverse fusion primer consisted of the Ion primer adapter trP1 at the 5’ end followed by the 806R primer. The primers produce a V4 product insert sequence of approx 250 bp which gives adequate resolution for taxonomic analysis [35]. PCR was performed in 25 μl vol and included: 2 μl (7.5 μM concn) of forward and reverse primers, 12.5 μl of Hot Start Taq 2X Master Mix (New England BioLabs Inc., Ipswich, MA., USA), 10 mM Tris-HCl, 2.5 μl of 10X bovine serum albumin (400 ng/μl final concn), and 2 μl of sample DNA. Thermal cycle conditions were 95°C for 3 min for initial denaturing step, followed by 30 cycles of 95°C for 30 s, 50°C for 1 min, and 72°C for 1 min. PCR products were checked on a 2% agarose gel for correct product size formation (approx 350 bp) and quantified using GelAnalyzer software. PCR products were then combined into an approximately equimolar mixture. The product mixture was size selected by first using a 2% agarose gel selection, (E-gel Size-Select 2%, Life Technologies, Grand Island, NY, USA) followed by an additional purification step with AMPure XP DNA magnetic beads (Beckman Coulter, Danvers, MA) according to manufacturers protocol. The final size-selected mixture was quantified using a Qubit 2.0 fluorometer (Life Technologies, Grand Island, NY, USA). Template preparation was performed using the Ion PGM OT2 400 kit according to manufacturer’s protocol with the Ion OneTouch 2 system, followed by sequencing with the Ion Torrent PGM using the 400 bp sequencing chemistry and either a 314v2 or 316v2 chip (Life Technologies, Grand Island, NY, US).

### Data processing and analysis

Quality filtering of sequences was performed using MOTHUR [36]. An average q score of 25 and a 25 bp window, with a max of 8 bp homopolymer and min read length of 150 bp and one bp difference in primer sequence were used as filtering criteria. Barcodes were used to assign reads to samples, and sequence data from different runs was concatenated into a single file for further analyses. Sequences were clustered into operational taxonomic units (OTU) based on 97% similarity using USEARCH global search algorithm [37] against the Greengenes release 13_8 97% OTU database [38]. Ribosomal gene copy number were estimated using picrust [39].

### Statistical analysis

All statistical calculations were performed using R [40]. Bray-Curtis distance and Principal Coordinate Analysis was performed using the package ‘vegan’ in R with relative abundance OTU table. Also, Beta-dispersions metrics, Shannon index, and PERMANOVA were calculated with the ‘vegan’ R package [41]. Prior to correlation analysis of families or OTUs with time, the taxa table was transformed using the centered log ratio (clr) to account for the compositional nature of the data [42], zeros were handled by adding 0.5 psuedo count to all OTU table data points to facilitate clr transformation [43]. Significance of correlation were corrected for multiple comparisons using the false discovery rate (fdr) adjustment [44]. Hierarchical clustering was performed using log_2_ transformed family abundances and the Ward2 algorithm [45].

### Accession numbers

The sequencing files are deposited in the NCBI Sequence Read Archive (SRX2353374-SRX2353438).

## Results

### DNA sequence recovery and classification

Ion Torrent sequencing from the two runs yielded a total of 1,440,171 usable reads after quality filtering with an average length of 247 bp. The 24 samples multiplexed in the first sequencing run (314v2 chip) generated 210,367 sequences, with 159,381 (75.7%) classified to an OTU at the 97% similarity threshold using Greengenes release 13_8 97% OTU [38]. The 41 samples multiplexed in the second sequencing run (316v2 chip) generated 1,229,804 sequences, with 836,521 (68%) classified at the 97% threshold. A total (both sequencing runs) of 995,902 sequences were clustered to 3422 OTUs with a mean of 15,321 sequences per sample. The total reads unclassified using Greengenes (97% threshold), after quality filtering, was 30.8%. The 314v2 run generated a total of 2,151 OTUs with an average count of 6,641 (min 2,839, max 15,593), and the 316v2 run generated a total of 2,880 OTUs with an average count of 20,403 (min 2,364, max 127,293). Sequencing results and metadata for individual samples are detailed in S1 Table.

For samples that were collected immediately after defecation and homogenized (S1 Table, regular samples from Loranger and Hammond Farms), there were 10 Phyla observed with a mean abundance > 0.1%. The relative percentages of phyla observed were the *Bacteroidetes* (34.2%), *Verrucomicrobia* (32.5%), *Firmicutes* (14.9%), *Fibrobacteres* (11.3%), *Spirochaetes* (5.2%), *Tenericutes* (1.1%), *Cyanobacteria* (0.3%), *Proteobacteria* (0.2%), *Euryarchaeota* (0.1%), *Lentisphaerae* (0.1%). Additionally, the phyla *SR1*, *Actinobacteria*, *Planctomycetes*, *Eluimicrobia*, *AD3*, and *Acidobacteria* were occasionally detected with relative abundances of less than 0.1%. The relative abundances of phyla for all samples and sample types are included as S1 Fig.

### Reproducibility of microbiome data from surface sampling or homogenization of horse manure, objective(i)

**Fig 1:**
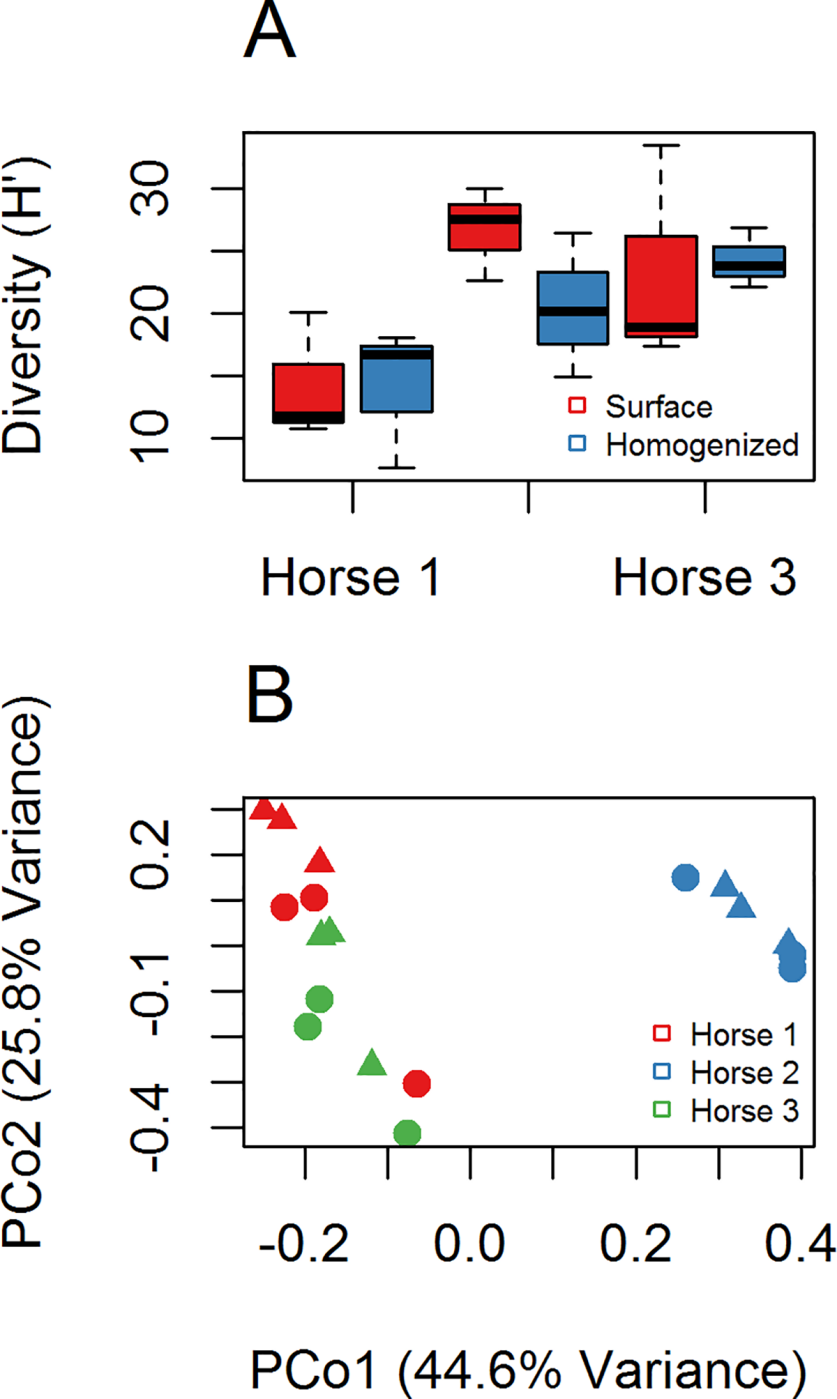
Changes in microbial composition from surface sampled and homogenized horse manure. (A) Boxplot of Shannon diversity (H’) between surface sampled and homogenized samples. (B) PCoA plot of community composition using Bray-Curtis dissimilarity and 97% similarity OTUs. Triangles (▲) are surface samples of manure, circles (●) are homogenized manure samples.

To assess if homogenization of samples reduces variability in comparison to surface sampling, we sampled freshly deposited horse manure pats from three separate horses. We found no effect of sampling method on Shannon diversity (Fig 1A) estimates using ANOVA (*p = 0*.*455*), but diversity differences between horses were significant (*p = 0*.*0012*). The assumption of homogeneity of variances of Shannon diversity was examined using Bartlett’s test, and was not significant (*p = 0*.*08*), but the low *P*-value suggests that surface sample diversity may be more variable than homogenized.

**Fig 2:**
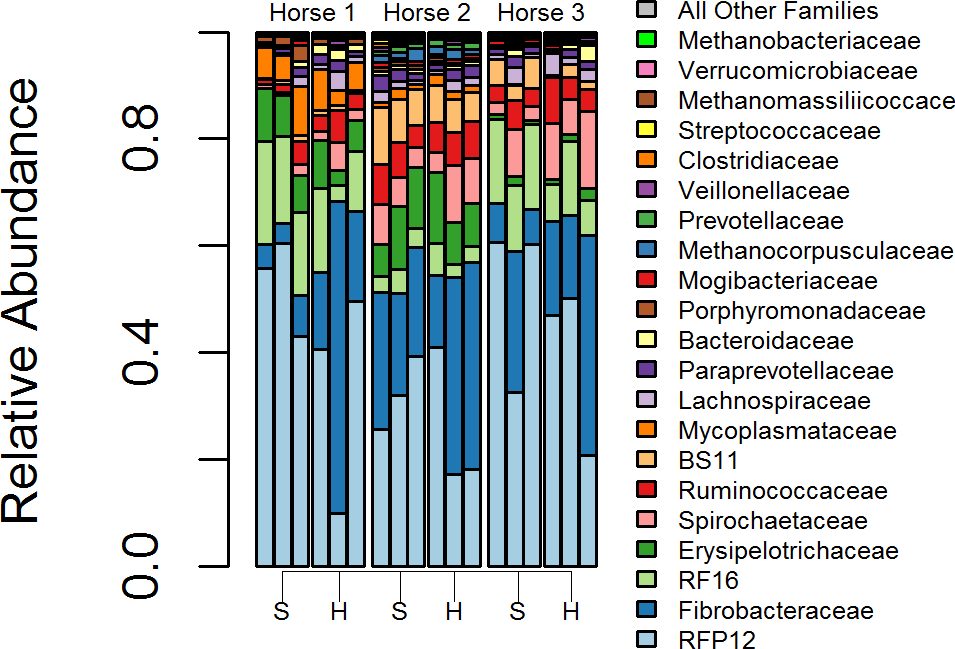
Family-level composition of surface sampled (S) and homogenized (H) manure piles from three different horses.

We evaluated changes in community composition between surface and homogenized samples first using a distance based permutation test (using 97% OTU Bray-Curtis distances, betadisper function in ‘vegan’ package) for homogeneity of variances [46] with ANOVA, which was not significant (*p = 0*.*175*). This test examines if the dispersion around centroids are greater with different treatments. Principal coordinates analysis (PCoA) of surface vs. homogenized samples support this result as dispersion between different samples did not appear visibly greater in surface or homogenized samples (Fig 1B). We utilized PERMANOVA to assess if there were differences between the centroids in surface and homogenized samples [47] with horse by method (surface vs homogenized) as the model. The effect of sampling method was significant in the model (*R*^2^ = 0.073, *p = 0*.*010*); however, the effect of horse was far greater (*R*^2^ = 0.629, *p = 0*.*001*), while no significant interaction was found (*p = 0*.*271*). The findings were supported by visual inspection of the PCoA plot (Fig 1B). Changes in microbial composition at the 97% OTU level and at the Family-level between surface and homogenized samples were examined using ALDEx2 [48], and no significant differences were found at either taxonomic level, also see Fig 2. The weak influence of sampling method on community dissimilarity and lack of significant taxonomic shifts between surface and homogenized samples relative to different horses suggest the sampling method to be a weak influence in this study.

### Changes in horse manure microbiome with time, objective(ii)

**Fig 3:**
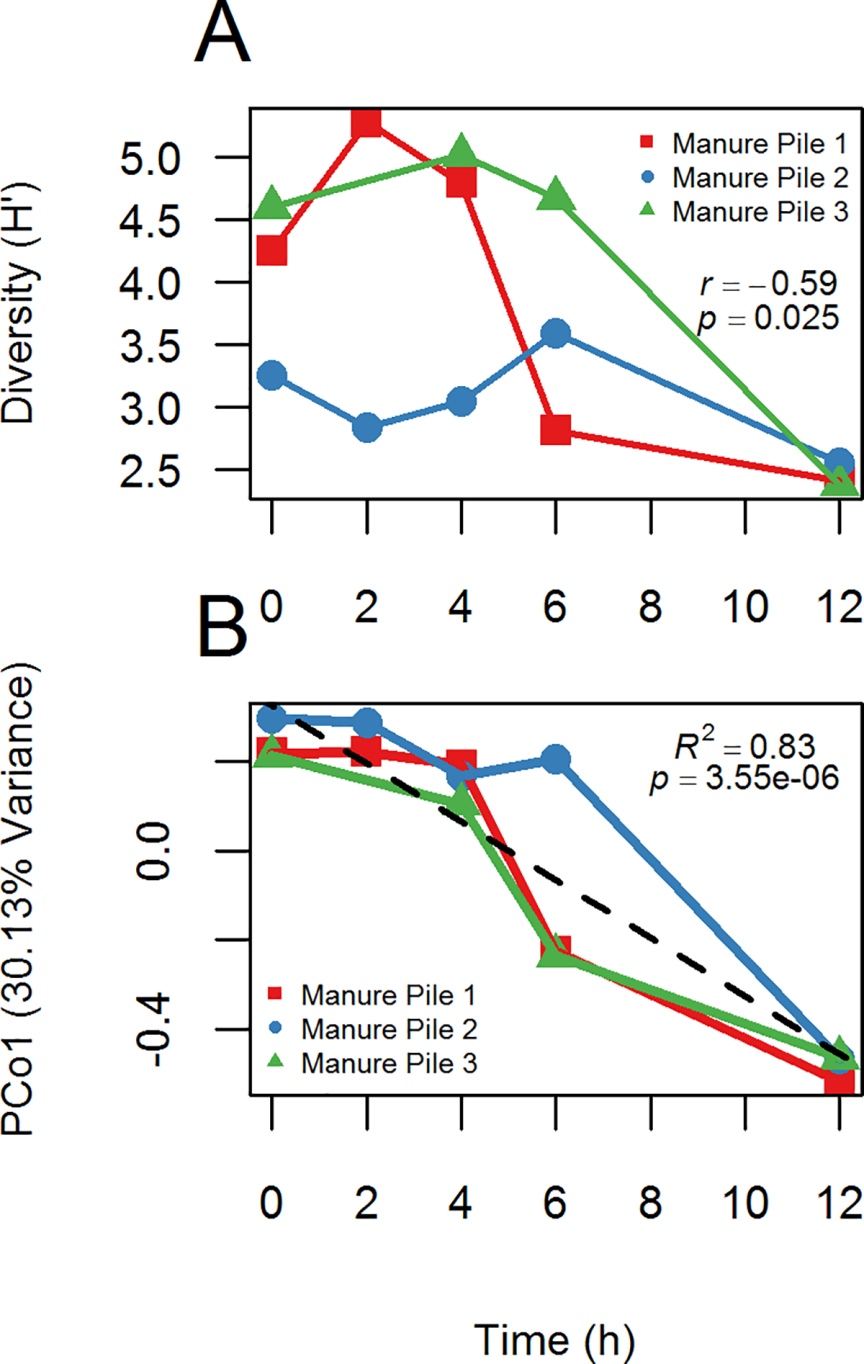
Changes in microbial composition with time. (A) Changes in Shannon diversity (H’) with time. (B) Plot of community composition (PCoA, PCo1) changes with time using Bray-Curtis dissimilarity and 97% similarity OTUs.

Changes in alpha diversity with time were measured using Shannon diversity (H’) as a function of time (Fig 3A). All three manure piles had peak diversity observed at intermediate time points, which decreased sharply by 12 h, resulting in a significant negative correlation (*R* = -0.59, *p = 0*.*025*). PCoA using Bray-Curtis dissimilarity with 97% OTUs (Fig 3B) was performed and the first six coordinates were retained. A linear model was constructed with time as the independent variable and the six PCo’s as dependent variables. Only the first principal coordinate was significantly correlated with time (*R*^2^ = 0.83, *p = 3*.*5e-06*), indicating time as strong predictor of microbial composition in the manure piles. Additionally, the effect of time and horse (source of manure pile) were both significant (*R*^2^ = 0.265, *p = 0*.001 and *R*^2^ = 0.329, *p = 0*.*001*, respectively) using PERMANOVA (adonis function, R vegan package).

### Identification of bacterial families increasing or decreasing with time, objective(ii)

**Fig 4:**
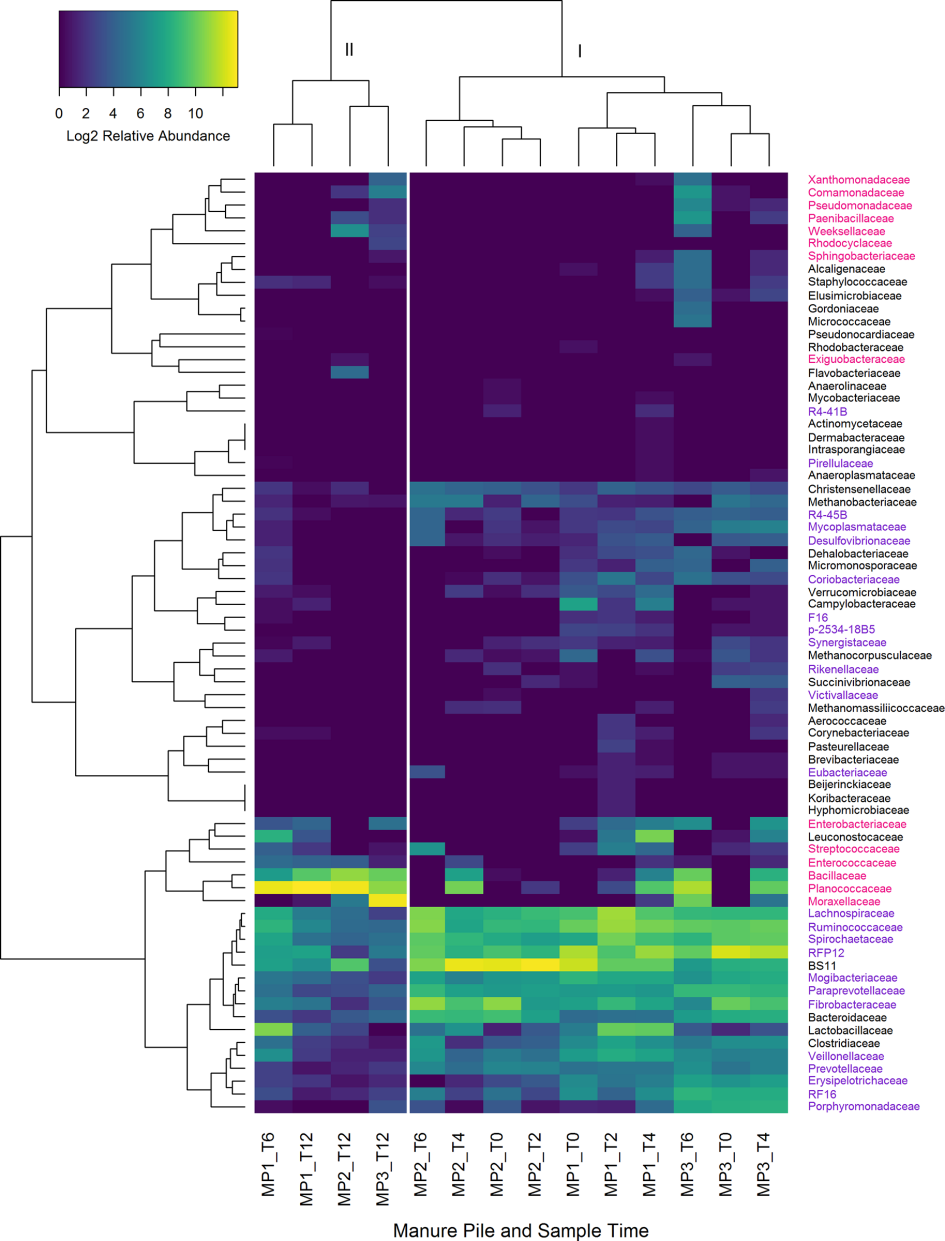
Heat map and hierarchical clustering of manure piles. Samples clustered by time and families that increased in abundance with time (magenta labels) and decreased with time (purple labels). Individual manure piles (MP1-3) are identified by time sampled post defecation.

Families of bacteria that either increased or decreased with time were determined using two methods. First, Pearson’s correlation using centered-log ratio transformed family abundance data with false discovery rate (fdr) adjustment [44] identified three families that increased with time, *Bacillaceae*, *Planococcaceae*, and *Enterococcaceae*, all of the class *Bacilli* (Table 1). The genera that increased within the family *Bacillaceae* were *Anaerobacillus*, *Anoxybacillus*, *Bacillus horikoshii*, and *Bacillus muralis*. Within the family *Enterococcaceae*, *Enterococcus*. Within *Planococcaceae*, *Lysinibacillus*, *Rummeliibacillus*, and *Solibacillus* increased with time. *Mogibacteriaceae* and *Ruminococcaceae* both of the class *Clostridiales*, significantly decreased with time. For the second test, we used Pearson’s correlation with fdr adjustment, but searched for correlation between families and the first principal coordinate from the PCoA, which is highly correlated with time (Table 1, and Fig 3B). Families that increased with time using the PCoA have a negative *R* value, since the correlation is to PCo1, and not time directly (see Fig 3B). Families that did not have significant correlation to either time or PCo1 were excluded from Table 1. Families that were found to increase with time using PCoA were *Flavobacteriaceae*, *Weeksellaceae*, *Sphingobacteriaceae*, *Bacillaceae*, *Exiguobacteraceae*, *Paenibacillaceae*, *Planococcaceae*, *Enterococcaceae*, *Comamonadaceae*, *Rhodocyclaceae*, *Enterobacteriaceae*, *Moraxellaceae*, *Psuedomonadaceaem*, and *Xanthomonadaceae*. Hierarchical clustering of manure piles separated samples into two major clusters (Fig 4), with cluster II having all of the manure pile communities at 12 h, and one of the 6 h samples. Within cluster I, three sub clusters were present, and were separated by horse (manure pile). Hierarchical clustering of microbial families clustered together by taxa that increased with time and those that decreased with time.

**Table 1:** Families associated with shifts in fecal microbiome over time.

Taxonomic classification				change w / time	Family vs. time (h)	Family vs. PCo 1
*Phylum*	*Class*	*Order*	*Family*		Pearson's R (p value^a^)	Pearson's R
*Actinobacteria*	*Coriobacteriia*	*Coriobacteriales*	*Coriobacteriaceae*	(-)	-0.525 (0.217)	0.398*
*Bacteroidetes*	*Bacteroidia*	*Bacteroidales*	*p-2534-18B5*	(-)	-0.538 (0.203)	0.43*
			*Paraprevotellaceae*	(-)	-0.558 (0.183)	0.709***
			*Porphyromonadaceae*	(-)	-0.143 (0.8)	0.573**
			*Prevotellaceae*	(-)	-0.541 (0.203)	0.646***
			*RF16*	(-)	-0.394 (0.446)	0.717***
			*Rikenellaceae*	(-)	-0.587 (0.159)	0.483**
	*Flavobacteriia*	*Flavobacteriales*	*Flavobacteriaceae*	**(+)**	0.359 (0.479)	-0.661***
			*Weeksellaceae*	**(+)**	0.572 (0.178)	-0.499**
	*Sphingobacteriia*	*Sphingobacteriales*	*Sphingobacteriaceae*	**(+)**	0.351 (0.479)	-0.69***
*Fibrobacteres*	*Fibrobacteria*	*Fibrobacterales*	*Fibrobacteraceae*	(-)	-0.687 (0.055)	0.838***
*Firmicutes*	*Bacilli*	*Bacillales*	*Bacillaceae*	**(+)**	0.854** (0.008)	-0.93***
			*Exiguobacteraceae*	**(+)**	0.707 (0.055)	-0.551**
			*Paenibacillaceae*	**(+)**	0.478 (0.286)	-0.769***
			*Planococcaceae*	**(+)**	0.813* (0.012)	-0.876***
		*Lactobacillales*	*Enterococcaceae*	**(+)**	0.809* (0.012)	-0.831***
	*Clostridia*	*Clostridiales*	*Eubacteriaceae*	(-)	-0.47 (0.286)	0.404*
			*Lachnospiraceae*	(-)	-0.686 (0.055)	0.595***
			*Mogibacteriaceae*	(-)	-0.761* (0.025)	0.495**
			*Ruminococcaceae*	(-)	-0.769* (0.025)	0.802***
			*Veillonellaceae*	(-)	-0.663 (0.068)	0.652***
	*Erysipelotrichi*	*Erysipelotrichales*	*Erysipelotrichaceae*	(-)	-0.344 (0.479)	0.601***
*Lentisphaerae*	*Lentisphaeria*	*Victivallales*	*Victivallaceae*	(-)	-0.3 (0.566)	0.414*
		*Z20*	*R4-45B*	(-)	-0.497 (0.252)	0.713***
*Planctomycetes*	*Planctomycetia*	*Pirellulales*	*Pirellulaceae*	(-)	-0.173 (0.784)	0.465*
*Proteobacteria*	*Betaproteobacteria*	*Burkholderiales*	*Comamonadaceae*	**(+)**	0.503 (0.248)	-0.665***
		*Rhodocyclales*	*Rhodocyclaceae*	**(+)**	0.315 (0.531)	-0.49**
	*Deltaproteobacteria*	*Desulfovibrionales*	*Desulfovibrionaceae*	(-)	-0.522 (0.217)	0.633***
		*Enterobacteriales*	*Enterobacteriaceae*	**(+)**	0.469 (0.286)	-0.392*
		*Pseudomonadales*	*Moraxellaceae*	**(+)**	0.703 (0.055)	-0.899***
			*Pseudomonadaceae*	**(+)**	0.196 (0.721)	-0.624***
		*Xanthomonadales*	*Xanthomonadaceae*	**(+)**	0.348 (0.479)	-0.592***
*Spirochaetes*	*Spirochaetes*	*Spirochaetales*	*Spirochaetaceae*	(-)	-0.661 (0.068)	0.561**
	*Synergistia*	*Synergistales*	*Synergistaceae*	(-)	-0.437 (0.346)	0.422*
*Tenericutes*	*Mollicutes*	*Mycoplasmatales*	*Mycoplasmataceae*	(-)	-0.385 (0.461)	0.594***
*TM7*	*TM7-3*	*CW040*	*F16*	(-)	-0.287 (0.585)	0.444*
*Verrucomicrobia*	*Pedosphaerae*	*Pedosphaerales*	*R4-41B*	(-)	-0.345 (0.479)	0.415*
* *	*Verruco-5*	*WCHB1-41*	*RFP12*	(-)	-0.686 (0.055)	0.858***

^a^ p-values adjusted using the Benjamini-Hochberg procedure

*significant at alpha = 0.05

** <0.01

*** <0.001

### Sampling stalled horses versus fresh droppings, objective(iii)

**Fig 5:**
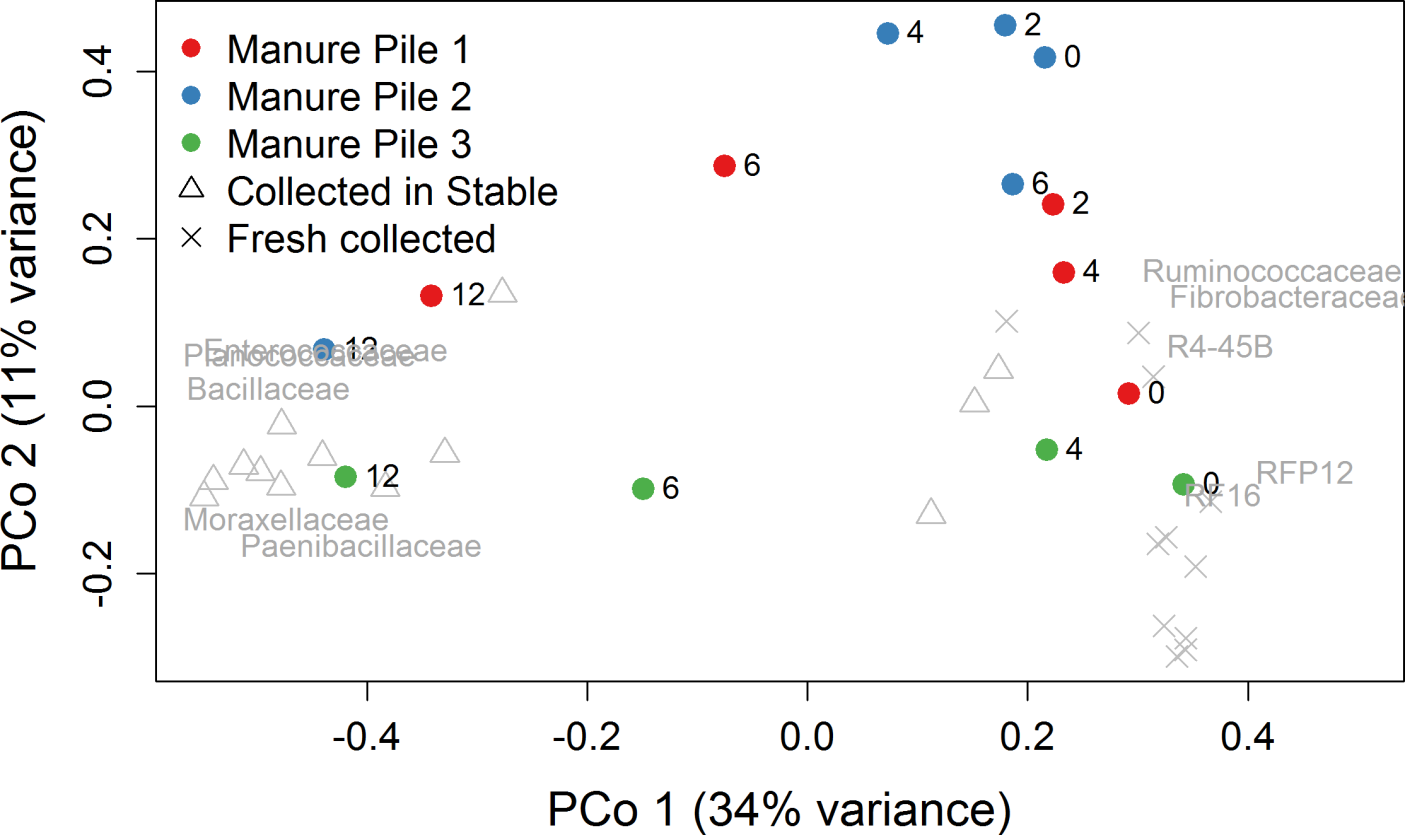
Principal coordinates plot of samples collected from visually observed fresh dropping and collected in pre-cleaned stalls. Samples from the time series study are also included, represented by colored circles. The 10 families with the strongest correlation to PCo1 are ordinated.

Principal coordinates analysis of samples collected from visually observed fresh droppings (11 from Hammond and Loranger farms, and 13 samples collected in pre-cleaned stalls with horses at the Folsom farm (S1 Protocol) revealed a large separation between the Folsom and Hammond/Loranger (stalled vs. freshly collected samples) along the first principal coordinate (Fig 5). Samples from the time series study were included in the analysis, and the 12 h samples clustered with the Folsom stalled samples. The Folsom samples clustered separately from Hammond and Loranger samples. The Hammond and Loranger samples clustered with the initial time points (0, 2, and 4 h) from the time series samples.

### 16S copy number and bloom taxa

Using picrust’s inferred 16S rRNA gene copy numbers, OTUs increasing with time in fecal pellets had an average rRNA gene copy number of 9.84 (± 0.69), while all detected OTUs in fresh feces had an average of 3.94 (± 0.69) copies per genome.

In comparison to previously reported horse fecal microbiome studies (see Supplemental Information, S2 Table, for data compiled from other studies), our study found *Bacteroidetes* to be the most abundant phylum (34.3%). We can find only two other NGS 16S amplicon studies that report *Bacteroidetes* as the most abundant [15,49]. Additionally, we found *Verrucomicrobia* to be the second most abundant (32.5%), which is also the highest proportion of *Verrucomicrobia* reported in any horse fecal microbiota study, although two previous studies found proportions of *Verrucomicrobia* around 27% [12,18]. An additional study found mean *Verrucomicrobia* proportions at 10%, but reported individual horse fecal samples as high as 43.1% [50]. Interestingly, the two studies that observed similar proportions of *Verrucomicrobia* as our study found *Firmicutes* as the predominant phyla present (36–69%), and *Bacteroidetes* in far less proportions (0.9–9.9%) than we observed. Our study found *Firmicutes* to be the third most abundant phyla (14.9%). In contrast, The majority of equine fecal microbiome NGS studies found *Firmicutes* as the most abundant phyla [12,15,17,18,51–54]. Relative abundances of *Bacteroidetes* and *Firmicutes* in horse fecal material determined using 16S rRNA amplicons has previously been shown to be DNA extraction method dependent [20], and the method reported to recover a greater *Bacteroidetes* percentage in that study (MoBio PowerFecal®) was similar to the method used in our study (MoBio PowerSoil®). Also notably different in our findings are *Fibrobacteres* having a mean proportion of 11.3%, which is the highest reported of all studies compared (S2 Table). Although the phyla observed in this study have been previously reported in afore-mentioned studies, it is clear that the proportions we observed are different. This wasn’t surprising, given the primer selections of almost all other previous studies were different than the primers used here (V4, 515f/806r, see S2 Table for comparisons of primers used in other studies). A recent study by Ericsson *et al*.[14] examining the microbiome along the equine gastrointestinal tract did utilize the same primer pair as our study, and comparison of microbial phyla from dorsal colon luminal contents in that study with freshly collected feces from our study were more similar than those using different primer pairs (S2 Table). Our selection of the V4 primers 515f/806r was based upon the reported nearly universal coverage of archaea and bacteria [33,55], and Earth Microbiome Project protocols [19]. The differences introduced due to primer bias between our study and that of others is especially highlighted in the high proportions of *Verrucomicrobia* we observed, which has been shown to be greatly underestimated with commonly used 16S primer pairs, but not when using the 515f/806r primers [56]. More recently, using metagenomic data in comparison to 16S rDNA amplicon data, it has been shown that 16S rDNA surveys using the 357-926R primer pair could miss up to 22% of the 16S rDNA sequences in the metagenomes surveyed [57], and even the best primer combination (515f/806RB) missed 9.6% of the 16S diversity from the same metagenomic dataset. While primer bias doesn’t necessarily exclude the usefulness of 16S rDNA amplicon based studies testing specific hypotheses, it does make inter-study comparisons non-trivial when different primer pairs are used. Given the broad range of the 515f/806r primer pair, and the high correlation of the V4 16S rRNA region (as well as V5 and V6) with phylogeny in comparison to V1, V2, V3, V7, and V8 regions [58], we would recommend this primer pair for future 16S rRNA based surveys of the horse fecal microbiome.

Comparison of sampling methodology, surface sampling vs. homogenization of samples, did not show significantly different results in this study. Though not significant, the diversity appeared more variable in surface sample replicates, in agreement with other findings, which highly recommend homogenization of fecal samples [26]. The sample size for comparing sampling methods was small in this study, but did not suggest a large effect on community composition and diversity. The only significant differences were between individual horses, which was expected, suggesting that either sampling method is appropriate, and comparable with one another.

When comparing fecal microbiome composition with time, *Bacillaceae*, *Planococcaceae*, *Enterococcaceae*, *Enterobacteriaceae*, and *Moraxellaceae* were found to be more abundant in all 12 h samples, with *Planococcaceae* and *Moraxellaceae* not detected in fresh samples. All of these families are either aerobes, facultative anaerobes, or aerotolerant anaerobes. Additionally, *Paenibacillaceae*, *Comamonadaceae*, *Rhodocyclaceae*, *Pseudomonadaceae*, *Xanthomonadaceae*, *Flavobacteriaceae*, *Weeksellaceae*, and *Sphingobacteriaceae* were also found to be more abundant at 12 h, but were not detected in every aged sample. *Planococcaceae*, *Paenibacillaceae*, *Comamonadaceae*, and *Sphingobacteriaceae* were also found to increase with time in cow feces [59]. The study of Amir *et al*. [32] identified potential bloom taxa from human feces down to specific OTUs; however, these OTUs included *Pseudomonadaceae*, *Enterobacteriaceae*, *Planococcaceae*, and *Bacillaceae*, as also found in horse feces. *Mogibacteriaceae*, and *Ruminococcaceae* were the only families that decreased significantly with time using the clr with Pearson’s correlation (Table 1), both of which are anaerobes in the order *Clostridiales*. Wong *et al*. [59] found decreases in abundance of the classes *Coriobacteriia*, *Bacteroidia*, and *Clostridia*. Additionally, Choo *et al*. [27] also found decreases in relative abundance of *Ruminococcaceae* and *Lachnospiraceae* with human fecal samples held at room temperature and Roesch *et al*. [60] found decreases in *Bacteroides*, *Clostridium*, and *Ruminococcus* spp. in human fecal samples within 72 h at room temperature, and also noted increases in *Enterobacteriaceae*. Changes in fecal community composition has not been observed in all studies, notably in the Lauber *et al*. [61] and Carroll *et al*. [25], which did not find significant changes of fecal microbiota with time at room temperature. Lauber *et al*. [61] did not examine the composition of fresh samples, but that of three and 14 day old samples. Carroll *et al*. [25] did sample within the 24 h time frame, but noted that there were no significant changes. Wong *et al*. [59] examined changes in cow pat community composition over 57 days, with the first time point at 48 h and our study ended at 12 h, but our findings are similar in that the community composition shifted from predominantly anaerobic bacteria to those capable of living in an aerobic environment. These results are congruent with the large oxygen level shift from animal gut to open environment.

The pattern of shifts in alpha diversity with time was from intermediate, to highest diversity, then lowest at 12 h in this study. Initially the samples are representative of the fecal microbiome, but after exposure to the aerobic environment the bloom taxa begin to increase in abundance while the gut microbes are still present in high relative abundance as well, resulting in the highest observed diversity at intermediate time points. By 12 h, the bloom taxa became dominant, overwhelming the original community resulting in decreased diversity. The Wong *et al*. [59] cow pat study observed an initial decrease in diversity at 48 h, then it increased again until 29 days. They did not observe an initial increase in diversity as in our study, but this is likely due to the difference in sample timing. Also, our study did not observe an increase of diversity with time after the decrease, again, this is likely to differences in the timing of the studies.

The greatest compositional changes observed with time (beta diversity) varied between four to six hours in this study (Fig 4). Before six hours, all samples clustered most closely by individual horses. At 12 h all samples clustered together, including one six hour sample (Fig 4, MP1). Roesch *et al*. [60] found the shifts in community composition were greatest in the period from 12 h to 24 h in their study using human fecal samples; however, their study did not sample before 12 h, and was at room temperature, whereas temperature in this study was at 32°C on average, which may explain in part why the community composition in this study changed more rapidly.

The early increases in *Bacillus* spp. and *Paenibacillus* spp. is not surprising, given that these bacteria are thought to be inhabitants of the foregut of non-ruminant animals (i.e., stomach, duodenum, jejunum, and ileum). For example, Tam *et al*. [62] found that Bacillus spores fed to mice were able to germinate and re-sporulate within the intestines. Studies in rabbits demonstrated that *Bacillus* spp. may potentially benefit the host as a gut symbiont [63], and human feces contains typically ~ 10^4 Bacillus spores per g [64], which suggests that Bacillus are actively growing in intestines, and not a contamination artifact from ingestion. In this study, we find that fresh manure is potentially an important habitat for Bacilli. Additionally, the *Bacillales* that were detected as bloom contaminants in this study were all inferred to have high 16S rRNA gene copies in their genomes, which has been demonstrated to confer a shorter lag time and a higher μ_max_ [65,66], with population maximum growth increasing directly proportional with 16S rRNA copy numbers [67]. Although this study did not examine whether or not Bacilli or other bloom taxa were present in the upper digestive tract of the specific horses whose feces was studied, we did examine 16S rDNA amplicon data from Ericsson *et al*. [14] available through NCBI SRA (PRJNA322656) and found that their samples contained bloom taxa in the upper digestive system of healthy horses (data not shown), supporting the hypothesis that these taxa were already present in fresh feces and were able to rapidly take advantage of the new environment, i.e. aerobic conditions, post defecation.

In this study a closed reference database approach was used to identify 16S amplicons, with the majority of sequences matching reference sequences at 97% similarity or greater. The unclassified sequences were between 24% and 32% in each sequencing run, which is lower than the unclassified OTU rate in other studies of the horse gut targeting the V4 region [17,54], but still suggests that there is much undescribed diversity within the guts of horses. This study was initialized in large part to insure that samples collected by different individuals from different geographical areas (crowdsourced) were not contaminated due to less than optimal sampling (i.e., collection of non-fresh feces) or holding conditions (e.g., storage, transport). We also wanted to examine the possibility of sampling horse fecal samples directly from stall floors. In this study, which utilized the same sample processing once in lab, we find that equine fecal samples post defecation change in microbial composition rapidly (in horse natural horse barn-stable conditions), which can be characterized by a bloom in aerobic taxa that have on average more 16S rRNA gene copy numbers per genome. Currently there are multiple equine projects that are turning to crowdsourcing for sample collection, and this study may help researchers make informed decisions when screening samples for integrity. This study is a step forward in that direction of standardizing horse fecal microbiome studies, but optimally a central lab resource is desirable to reduce inter-lab bias for the horse gut microbiome.

## Supporting information

S1 Fig Relative abundance of Phyla by sample groups. (A.) surface vs. homogenized samples, (B.) ‘regular’ samples (C.) Stall collected samples from Folsom, (D.) time-series post-defecation samples.
(TIFF)

S1 Table Sample information. (XLSX)

S2 Table Comparison of Phyla relative abundances and methods from previous studies. Comparison of relative abundances of phyla from other studies, DNA extraction methodology, PCR primers used, and DNA sequencing technology.
(XLSX)

S1 Protocol Sampling Protocols. Sampling methodology for different objectives (i-iii) with graphical flow-chart.
(PDF)
