# Supplementary figures and images for: Rapid regrowth and detection of microbial contaminants in equine fecal microbiome samples
Source: PLoS One. 2017 Nov 1;12(11):e0187044. doi: 10.1371/journal.pone.0187044 (PMC5665523; doi:10.1371/journal.pone.0187044)

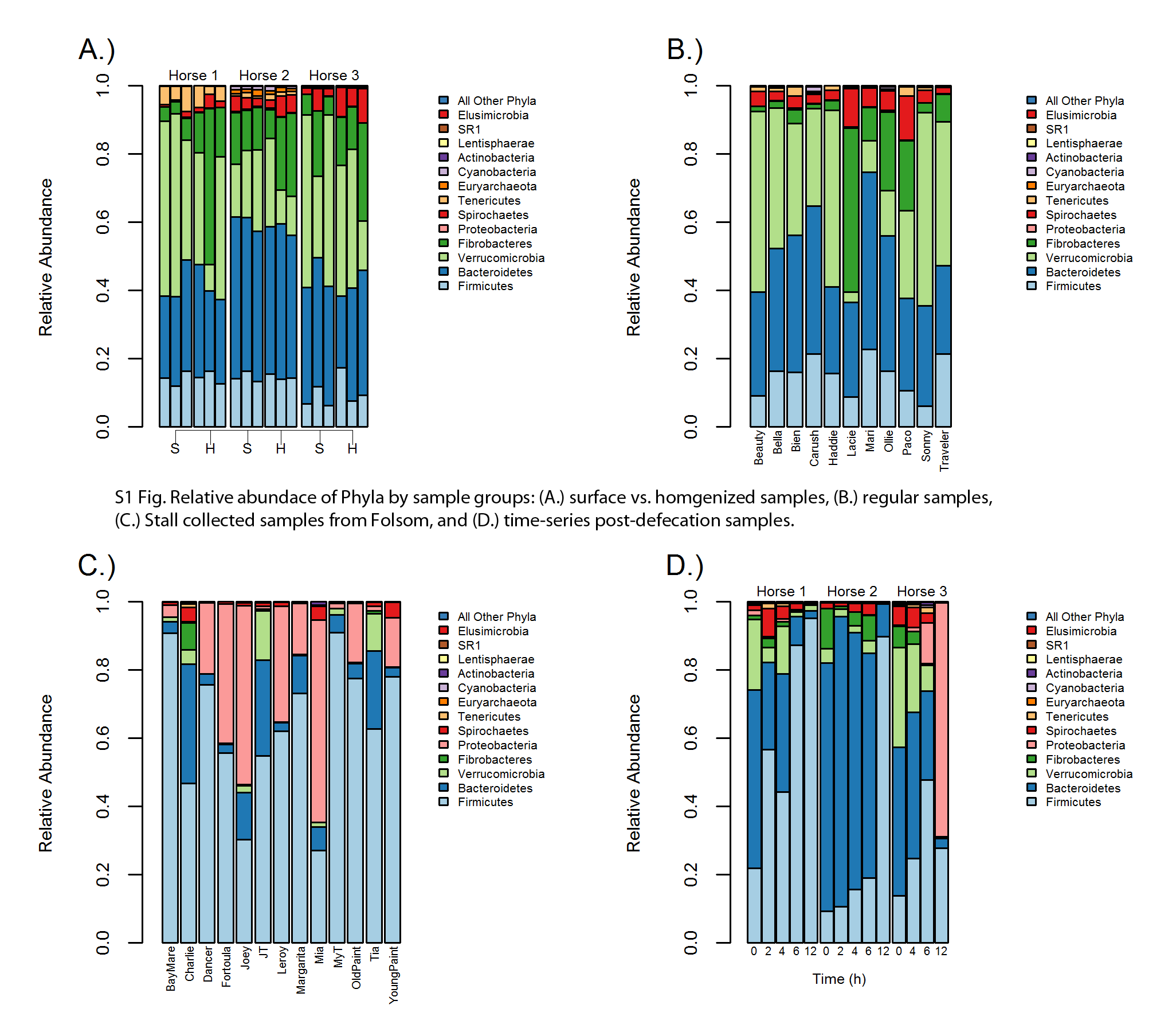

Supplement: S1 Fig — (A.) surface vs. homogenized samples, (B.) ‘regular’ samples (C.) Stall collected samples from Folsom, (D.) time-series post-defecation samples. (TIFF) [file pone.0187044.s001.tiff]
