# Supplementary material for: Rapid regrowth and detection of microbial contaminants in equine fecal microbiome samples
Source: PLoS One. 2017 Nov 1;12(11):e0187044. doi: 10.1371/journal.pone.0187044 (PMC5665523; doi:10.1371/journal.pone.0187044)
Supplement: S1 Protocol — Sampling methodology for different objectives (i-iii) with graphical flow-chart. (PDF) [file pone.0187044.s004.pdf]

S2 Sampling Protocol

This study had three basic objectives (i-iii):

i.) Examine if sampling either from the surface of a horse fecal pellet pre-DNA extraction would affect the results of a NGS 16S rDNA amplicon library in comparison to homogenizing of a pellet before DNA extraction.
ii.) Determining if bloom taxa rapidly contaminate horse fecal samples, and also which taxa are candidate bloomers that can be used to identify compromised samples. This is especially important for crowdsourcing of horse fecal samples when improper storage conditions may occur.
iii.) Examine the possibility for collecting horse fecal samples directly from stalls which house individual horses as a method to rapidly collect many samples. This objective relates to objective ii.

Detailed Sampling methods: Flow-charts for each sampling procedure (i - iii) are provided below. For objective **(i)** three freshly deposited manure piles from three individual horses were visually observed at the time of deposition at the Loranger Farm, and sampling was performed immediately afterwards by taking small (approx 2g) scrapings from the exterior of a single pellet for 'surface' and placing into separate sterile 36oz whirl-paks (Nasco, Inc. Fort Atkinson, WI, USA). Following surface sampling, the remaining fecal pellet was placed into a whirl-pak for 'homogenized' sampling, and all samples were placed on ice and transported directly to a -20°C freezer until DNA extraction, for a total of 18 samples. After samples were thawed immediately before DNA extraction, 'homogenized' samples were homogenized by kneading the pellet inside the whirl-pak by hand for 1min, then DNA extraction proceeded as described in manuscript. For objective **(ii)** three fresh manure piles were marked off at time of deposition from three individual horses at the Loranger Farm and five samples were taken at timed intervals (14 samples, one lost in processing) from each manure pile. All samples were in shaded areas (barn). The average ambient temperature for the 12 hr period was 32ºC (stdev ± 3.6). At time of deposition (T0) an individual pellet from the manure pile was collected, immediately frozen, then processed using the homogenized sampling method. Additional samples were collected at 2, 4, 6, and 12 hrs (T2, T4, T6, and T12). To address objective **(iii)** 24 samples were collected, six samples from the Loranger Farm, five samples from the Hammond Farm, and 13 samples from the Folsom Farm in January of 2015. All samples from the Hammond and Loranger Farms were collected using the wait-for-the-drop-method followed by the homogenized technique. Samples collected from the Folsom Farm were collected from individual stalls that housed a single horse with a clay surface with wood shaving. The stalls had been cleaned within the previous six hrs by removal of horse and wood shavings, and addition of new wood shavings. Samples were processed using the homogenized sampling procedure.

Objective (i): Difference between surface and homogenized sampling

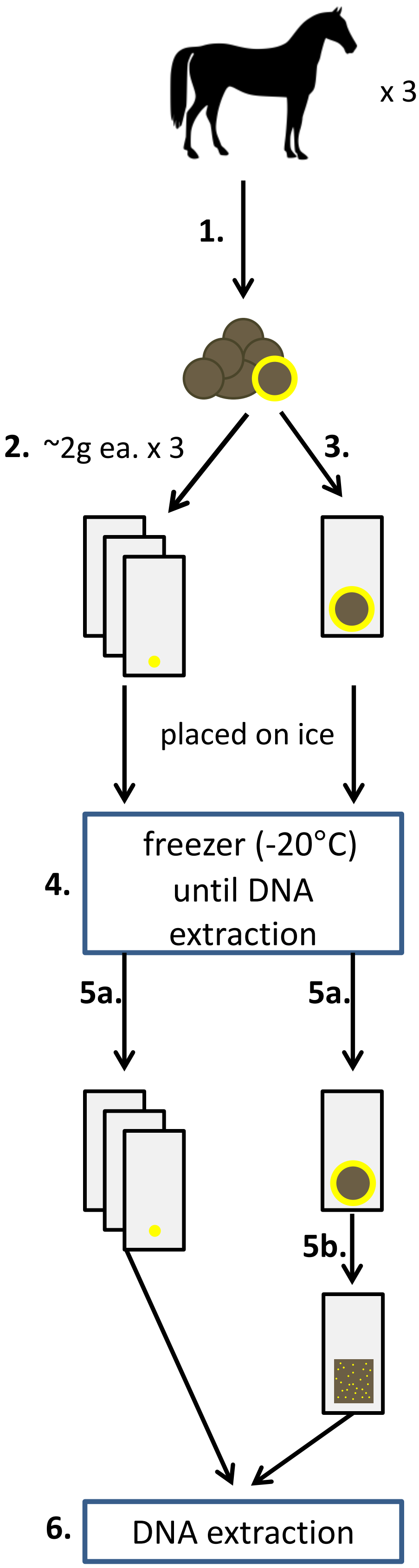

1.) Individual Horses were observed until a defecation event.

2.) Immediately after defecation, three samples were collected from the surface (yellow = pellet surface ~ 1cm) of a single fecal pellet by scraping ~ 2g using a sterile spatula each time placing into separate sterile 36oz whirl-paks, then on ice for transport to freezer.

3.) The remainder of the pellet sampled was placed in it's own 36oz whirl-pak, then on ice for transport to freezer.

4.) Samples stored at -20°C until DNA extraction.

5a.) Samples were defrosted, and processing began while still cold.

5b.) The single pellet collected in step 3 was homogenized by kneading within whirl-pak for 1min.

6.) DNA extraction was performed using MoBio Powersoil DNA Isolation kit (Mo Bio Laboratories Inc., Carlsbad, CA) according to manufacturers protocol.

Objective (ii): Time series sampling for 'bloom' taxa identification

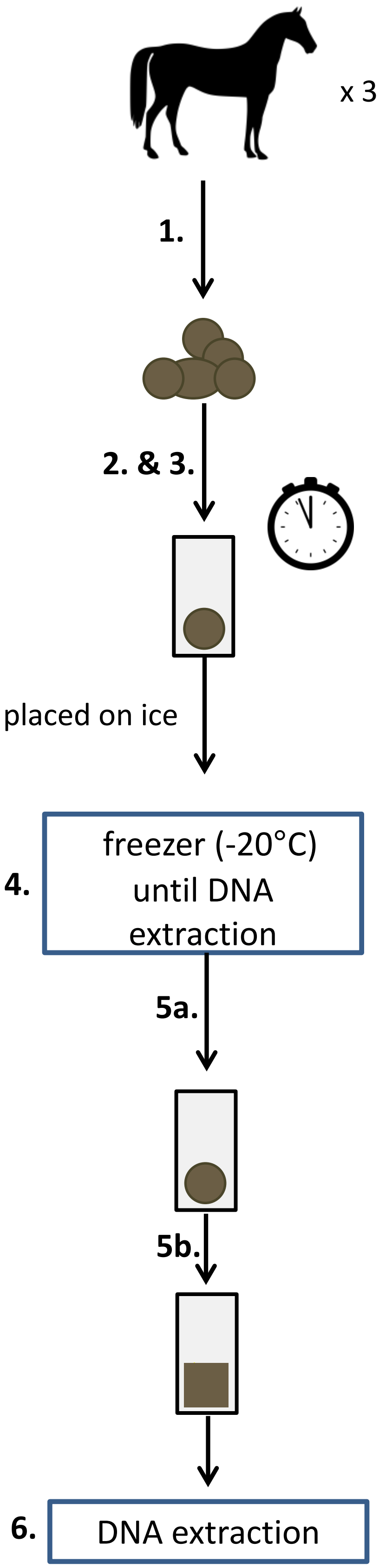

x 3 horses (i.e., 3 sets of samples)

1.) Individual Horses were observed until a defecation event, and fecal pellet was roped off to prevent horses from disturbing manure pile during 12 hr time-series collection.

2.) Immediately after defecation, a single fecal pellet was removed from the manure pile and placed into a sterile 36oz whirl-paks, then on ice for transport to freezer. This pellet was labeled as Time 0 (T0).

3.) The manure pile was sampled again at 2 hrs, 4hrs, 6 hrs, and 12 hrs, removing an individual pellet at each time point. Pellets that were on the bottom were avoided to avoid direct ground contamination.

4.) Samples stored at -20°C until DNA extraction.

5a.) Samples were defrosted, and processing began while still cold.

5b.) The single pellet collected was homogenized by kneading within whirl-pak for 1min.

6.) DNA extraction was performed using MoBio Powersoil DNA Isolation kit (Mo Bio Laboratories Inc., Carlsbad, CA) according to manufacturers protocol.

Objective (iii): Feasibility of sampling stalled horses without witnessing actual defecation event.

'Stalled' Fecal Sampling

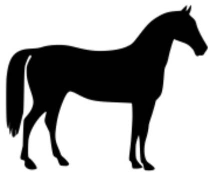

x 13 stalled horses, samples collected between 0 and 6hrs from time of defecation.

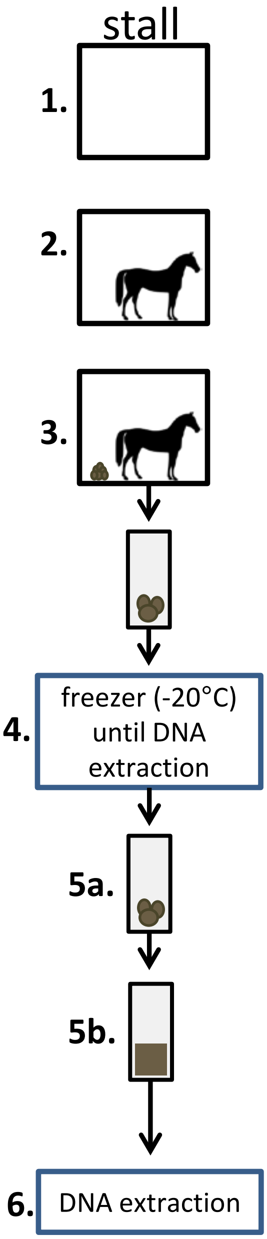

1.) As part of routine farm maintenance, horses as removed from stall and all manure is removed from stall (cleaning), including soiled wood shavings. New shavings are added.

2.) Horses are brought back into stall, as part of their normal routine.

3.) The stall is re-visited 6 hrs later to collect fecal pellets (a few fecal pellets from top of manure pile are placed into sterile whirl-paks, and placed on ice). Exact time of defecation (i.e., feces age) is unknown, but must be between 0 - 6 hrs of age at max.

4.) Samples stored at -20°C until DNA extraction.

5a.) Samples were defrosted, and processing began while still cold.

5b.) The pellets collected were homogenized by kneading within whirl-pak for 1min.

6.) DNA extraction was performed using MoBio Powersoil DNA Isolation kit (Mo Bio Laboratories Inc., Carlsbad, CA) according to manufacturers protocol.

'Fresh' Fecal Sampling

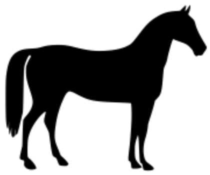

x 11 horses, samples collected immediately after defecation

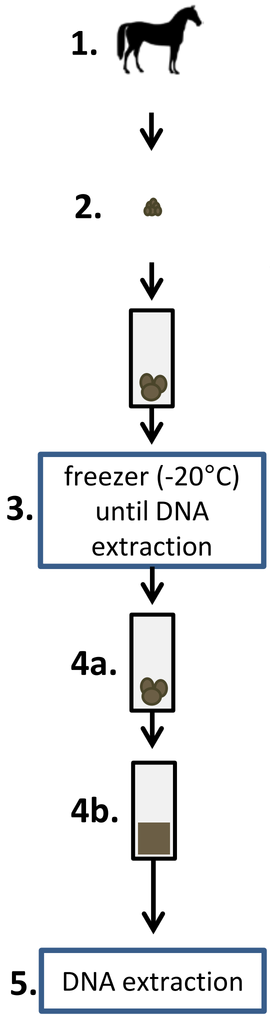

1.) Individual Horses were observed until a defecation event.

2.) Immediately after defecation, several pellets were collected from the surface and placed into a sterile 36oz whirl-pak, then on ice for transport to freezer.

3.) Samples stored at -20°C until DNA extraction.

4a.) Samples were defrosted, and processing began while still cold.

4b.) The pellets collected were homogenized by kneading within whirl-pak for 1min.

5.) DNA extraction was performed using MoBio Powersoil DNA Isolation kit (Mo Bio Laboratories Inc., Carlsbad, CA) according to manufacturers protocol.
